# New Onset Paroxysmal Atrial Fibrillation After Electroconvulsive Therapy

**DOI:** 10.7759/cureus.11717

**Published:** 2020-11-26

**Authors:** Michael R Hower, Chong Yang

**Affiliations:** 1 Psychiatry, Touro University of California - College of Osteopathic Medicine, Vallejo, USA; 2 Psychiatry, California Department of State Hospitals - Napa, Napa, USA

**Keywords:** ect, electroconvulsive therapy, atrial fibrillation

## Abstract

Electroconvulsive therapy (ECT) is a safe treatment for various psychiatric disorders. During an ECT treatment, an electrical stimulus produces a transient sympathetic response leading to changes in the cardiac rate and rhythm. Rarely, ECT treatment may precipitate atrial fibrillation (AF). In this case report, we present a 70-year-old man with schizoaffective disorder who developed paroxysmal AF after his 38^th^ ECT treatment. We review his risk factors for AF and propose a possible mechanism of its development. We also discuss potential treatment options to safely resume ECT treatments.

## Introduction

Atrial Fibrillation (AF) is a cardiac arrhythmia defined by abnormalities in the structure or conduction of the heart that promote abnormal atrial impulse formation and/or propagation [1]. It is detected by the absence of P waves and the presence of an irregular rate and rhythm on an electrocardiogram (EKG) [2]. The diagnosis is also classified by the duration of episodes. For example, the episode is “paroxysmal” when it begins suddenly and terminates within seven days [1].

The clinical presentation of AF may vary. A literature review indicates that up to 30% of patients with AF are asymptomatic. Otherwise, patients may present with a variety of symptoms, including palpitations, chest pain, dyspnea, fatigue, and more rarely, dizziness and syncope [3]. Regardless of the signs and symptoms, a cardiological assessment is necessary as AF is a clinically significant independent risk factor for stroke [4]. Therefore, patients should be evaluated and treated with their risk factors taken into consideration. Risk factors predisposing individuals to AF include increasing age, hypertension, obesity, diabetes mellitus, previous cardiovascular event, family history, European ancestry, alcohol use, and smoking [1]. The risk of AF is also higher in men than in women [5]. Finally, there are reports of new onset AF using antipsychotic medications, such as olanzapine and clozapine, likely due to their effect as an antagonist on muscarinic receptors in the heart [6-8].

The American Psychiatric Association supports the application of electroconvulsive therapy (ECT) as a treatment modality for major depression, mania, and schizophrenia. It is a safe medical procedure, and there are no absolute medical contraindications to ECT [9]. However, careful consideration is necessary for patients with cardiovascular disorders receiving ECT. The electrical stimulus produces a sympathetic response, including sinus tachycardia and transient EKG changes [10, 11]. This sympathetic response leads to increased oxygen consumption, blood pressure, and heart rate, which may cause additional stress on the heart [12]. Although ECT-induced AF is rare, this catecholamine surge may cause stretching of atrial fibers predisposing an individual to AF during treatment [13, 14]. In the following case report, we present a patient who developed atrial fibrillation after treatment with ECT.

## Case presentation

Our patient is a 70-year-old man hospitalized in a state hospital with a diagnosis of schizoaffective disorder, bipolar type. His psychotic symptoms include auditory hallucinations, disorganized speech, disorganized behavior, and paranoid delusions. His mood symptoms include mood swings, irritability, grandiosity, pressured speech, a flight of ideas, and impulsivity. He did not respond to multiple psychotropic medication trials during his hospitalization. Furthermore, he was a poor candidate for clozapine due to his constant refusals to obtain the necessary lab work. Therefore, he was referred for ECT treatment while remaining adherent to olanzapine 50 milligrams (mg)/day and lurasidone 160 mg/day.

The patient was treated on the Thymatron® System IV (Somatics, LLC, USA) with a pulse width of 0.5 milliseconds. Other treatment parameters include bilateral stimulus electrode placement, 1 milligram/kilogram (mg/kg) of methohexital as the anesthetic, and 1 mg/kg of succinylcholine as the muscle relaxant. Over the course of four months, he received 38 ECT treatments with partial relief of his psychotic and mood symptoms.

**Figure 1:**
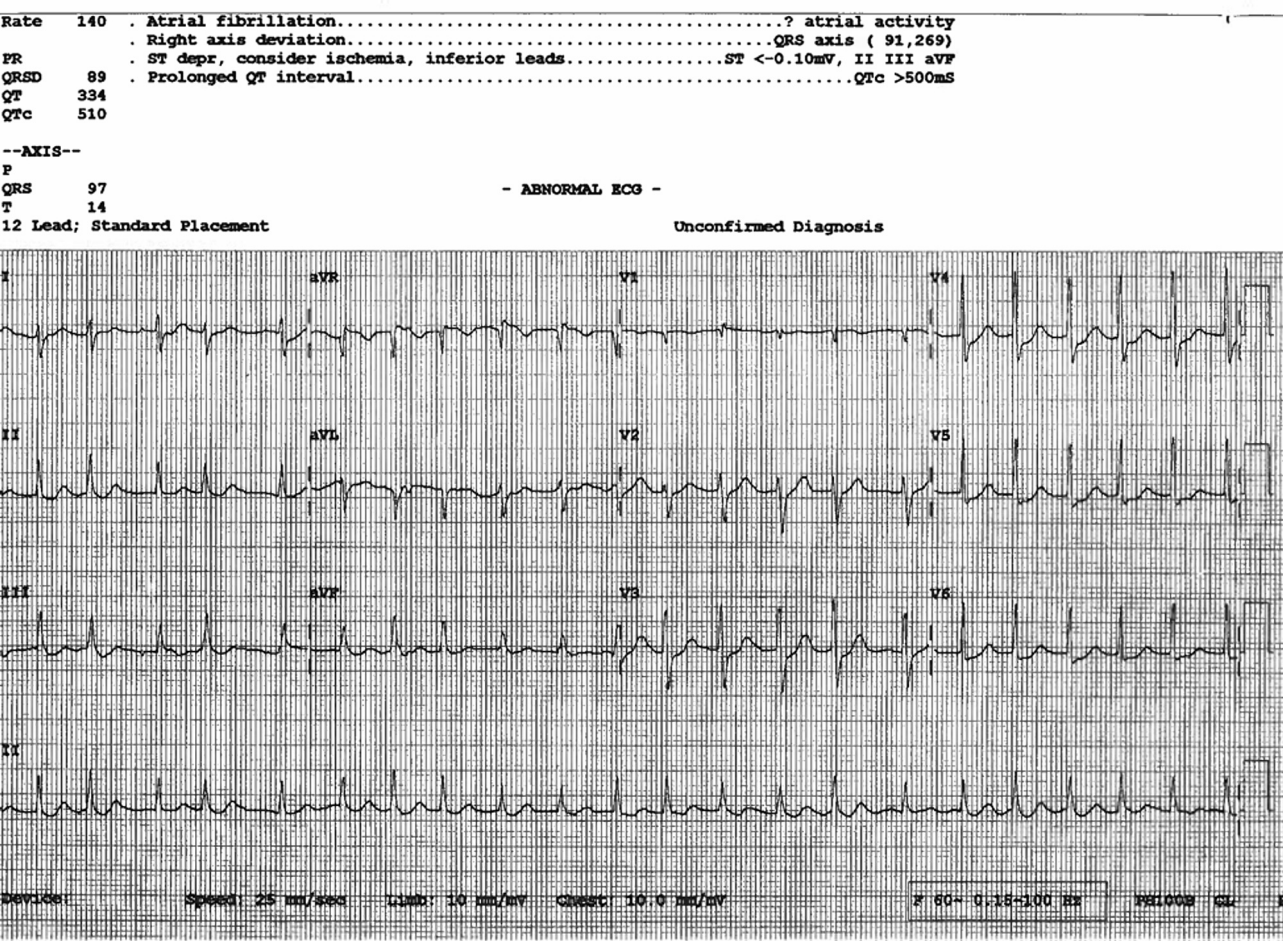
EKG after the 38th ECT treatment The EKG is characterized by the absence of P waves and the presence of an irregular rate and rhythm.

After his 38^th^ ECT treatment, the patient developed atrial fibrillation during the recovery phase. His heart rate reached 140 bpm, and an EKG confirmed the presence of atrial fibrillation (Figure 1). He did not report palpitations, fatigue, weakness, or dizziness. However, he was transferred to the emergency department for an additional medical workup. The patient remained asymptomatic upon transfer to the emergency department. His laboratory workup was unremarkable: complete blood cell count, serum electrolytes, blood urea nitrogen, creatinine, thyroid-stimulating hormone, cardiac enzymes, prothrombin time, and international normalized ratio are within normal limits. His EKG in the emergency department revealed a normal sinus rhythm with a rate of 68 bpm. He was observed for two hours and discharged back to the state hospital with a diagnosis of cardiac arrhythmia.

## Discussion

We presented a rare case in which a patient developed AF after treatment with ECT. We identified his age, gender, and antipsychotic medication (olanzapine) as his risk factors for AF. We propose that his risk factors and the sympathetic response with his ECT treatment precipitated his development of AF. Fortunately, the patient did not require immediate treatment due to his spontaneous conversion to normal sinus rhythm.

We reviewed the literature on the risks of continuing ECT for individuals with AF. Multiple case reports support the continuation of ECT treatment for individuals with AF [10, 13, 15]. Loeffler and Capobianco resumed ECT treatments in a healthy 46-year-old man with AF after administering a calcium channel blocker (diltiazem) and spontaneous cardioversion [13]. Other antiarrhythmic drugs such as beta-blockers may serve as therapeutic and prophylactic solutions. Labetalol was found to be superior to esmolol for protection against the increase in heart rate and blood pressure [14]. However, esmolol has a lesser impact on decreasing the seizure duration in ECT [14, 16].

In addition to antiarrhythmic medications, anticoagulation and cardioversion are considerations for the treatment of AF. Suzuki et al. reported a case study in which a 77-year-old woman developed an embolic stroke one day after her last ECT treatment [17]. To mitigate this risk, Petrides and Fink recommend anticoagulation prior to the continuation of ECT treatment [15]. It is important to note that anticoagulation is necessary prior to cardioversion to reduce embolization risk [18].

## Conclusions

In rare occurrences, patients develop atrial fibrillation during a course of ECT treatment. These patients should be assessed due to their increased risk of stroke. Furthermore, it is important to consider treatment of AF with antiarrhythmic medications, anticoagulation and/or cardioversion prior to the continuation of ECT treatment. These precautions minimize the risk of stroke so that they may safely resume ECT treatments.
